# The Logic of Fashion Cycles

**DOI:** 10.1371/journal.pone.0032541

**Published:** 2012-03-07

**Authors:** Alberto Acerbi, Stefano Ghirlanda, Magnus Enquist

**Affiliations:** 1 Centre for the Study of Cultural Evolution, University of Stockholm, Stockholm, Sweden; 2 Honors Academy and Department of Psychology, Brooklyn College, Brooklyn, New York, United States of America; 3 Department of Zoology, University of Stockholm, Stockholm, Sweden; Universita' del Piemonte Orientale, Italy

## Abstract

Many cultural traits exhibit volatile dynamics, commonly dubbed fashions or fads. Here we show that realistic fashion-like dynamics emerge spontaneously if individuals can copy others' preferences for cultural traits as well as traits themselves. We demonstrate this dynamics in simple mathematical models of the diffusion, and subsequent abandonment, of a single cultural trait which individuals may or may not prefer. We then simulate the coevolution between many cultural traits and the associated preferences, reproducing power-law frequency distributions of cultural traits (most traits are adopted by few individuals for a short time, and very few by many for a long time), as well as correlations between the rate of increase and the rate of decrease of traits (traits that increase rapidly in popularity are also abandoned quickly and vice versa). We also establish that alternative theories, that fashions result from individuals signaling their social status, or from individuals randomly copying each other, do not satisfactorily reproduce these empirical observations.

## Introduction

While some cultural traits, once introduced in a population, tend to become to varying degrees stable part of the cultural repertoire of that population, others exhibit peculiar volatile dynamics, commonly dubbed fads or fashions. Well documented examples are as diverse as skirt lengths [1], pop songs [2], first names [3]–[5], dog breeds [6], pottery decorations in the archaeological record [7], and keywords in academics vocabulary [8]. Such fluctuations in popularity are not mainly due to intrinsic characteristics of the traits–there seems to be nothing intrinsically advantageous about, say, wearing purple one year but not the next–but they are likely to reflect forces that are internal to cultural dynamics.

Since at least the 18^th^ century, fashions have been considered a product of social stratification [9]–[13]. According to such “status” models, a fashion arises because individuals of low social status copy those of perceived high status. When a trait becomes popular, however, high-status individuals quickly abandon the trait to differentiate themselves from low-status individuals. As a consequence, low-status individuals abandon the traits too, bringing the fashion cycle to an end (for recent computational models see [14]–[16]).

**Figure 1:**
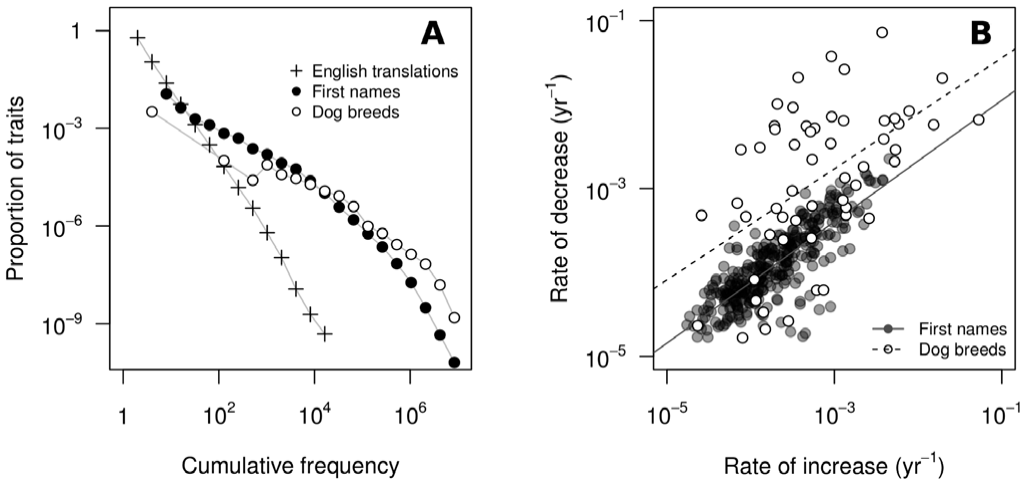
Empirical findings on fashion cycles. (A) Frequency distributions of cultural traits often follow power-law or log-normal distributions, i.e., the vast majority of traits remains very rare, while a small minority become very popular. Crosses: Number of times a foreign author has been translated into English (with permission from UNESCO's *Index Translationum*) [38]. Closed circles: number of times a first name has been given to a newborn in the U.S.A., 1880–2006 [32]. Open circles: number of dogs of 154 breeds registered with the American Kennel Club, 1926–2005 (courtesy of H. Herzog) [6]. (B) Correlation between the rates of increase and decrease in the popularity of U.S.A. first names (Pearson's 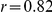, 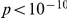, 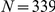, only names reaching a frequency of at least 0.1% are included, see [5], [32]) and dog breeds (Pearson's 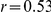, 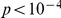, 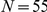).

Recently, however, a “neutral” model of cultural change [4] has been proposed as a more parsimonious explanation of fashions. In such a model fashions arise as by-products of individuals copying each other randomly (akin to changes in allele frequency in neutral genetic evolution [17]). The main appeal of this model is its simplicity, and the fact that it reproduces realistic turnover rates as well as empirical frequency distributions of cultural traits in several domains. These distributions closely approximate power-law or log-normal curves [2], [4], [8], meaning that only very few cultural traits become very common, while the vast majority remains rare (Figure 1A).

While the neutral model provides a powerful starting point to study cultural change [4], [18], there are many reasons to believe that fashions are not driven by random choices. Individuals express strong positive and negative preferences for cultural traits, and prefer to copy some models rather than others [19]–[21]. Status models recognize these factors, at the cost of postulating that high-status individuals are anti-conformist while everyone else is conformist. Status models also postulate a given subdivision between high- and low-status individuals. A model that could explain why some individuals have higher status would yield greater insight into fashion dynamics.

We present here an alternative model of fashion and fads that builds on our previous work on the effects of repeated cultural transmission on the frequency of cultural traits [22]–[24]. We study the possibility that fashions and fads arise because individuals can copy each other's preferences for cultural traits, in addition to the traits themselves. We call this the “preference model” of fashion. Because preferences determine which traits appear most attractive to individuals, they act as “regulatory” traits in the sense that are both socially learned and influence the outcome of social learning. This highlights an important difference between cultural and genetic evolution: in the former the rules of transmission (e.g., whether to copy or not) may be modified by the cultural process itself [22]–[25].

We first consider the coevolution of one cultural trait and the preference for such trait, showing that the the repeated cultural transmission of trait and preference is enough to generate a fashion cycle in which the trait first becomes popular and then disappears from the population. We then consider the simultaneous coevolution of many trait-preference pairs, showing that cultural transmission alone produces fashion dynamics exhibiting two key properties of actual fashion cycles: the power-law distribution of frequency of cultural variants mentioned above, and the finding that cultural traits that increase rapidly in popularity are also abandoned quickly, while slow increase in popularity correlates with slow decrease. This has been shown for first names in the U.S. and France [5], and here we report that it also holds for the popularity of dog breeds, based on an analysis of data on dogs of 154 breeds registered with the American Kennel Club, 1926–2005 (Figure 1B). Lastly, we show that the neither the neutral model of cultural change, nor models based on status can account for the same breadth of empirical data.

## Methods

### Model 1: One cultural trait

Our core idea is that preferences for cultural traits, besides influencing the adoption and abandonment of traits, are themselves cultural traits that can be adopted or abandoned through social learning. We start exploring this idea modeling the coevolution of one cultural trait and the preference for such trait. Individuals can be of one of four types: 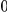, lacking both trait and preference; 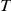, possessing the trait only; 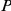, possessing the preference only; 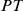, possessing both. Individuals meet randomly in pairwise social interactions in which one individual (the *observer*) may copy another (the *model*). The trait and the preference may be copied independently of each other. The probability that copying occurs between any two cultural types is 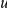, apart in the following cases:

- When the model has the trait, an observer with the preference is more likely to copy the model than an observer without the preference. We thus assume that copying between 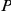 observers and 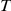 or 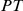 models, and between 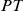 observers and 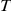 models, occurs with increased probability 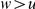.
- Copying between 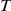 observers and 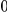 models occurs with increased probability 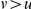, meaning that individuals with the trait, but without the preference, have a higher probability of abandoning the trait upon meeting individuals with neither trait nor preference.

Note that copying may result in the observer abandoning the trait or preference, if these are absent in the model.

Our aim is to track the frequency of the four cultural types 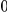, 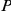, 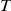, and 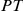 over time to understand the cultural dynamics generated by our assumptions. Taking into account all possible transitions between types (e.g., a 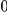 observer who meets a 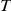 model becomes 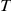 with probability 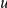), we arrive at the following equations (see Model S1, Figure S1, S2, S3, and Table S1 for details):
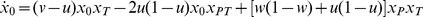
(1)

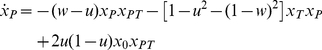
(2)

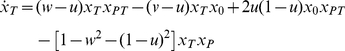
(3)

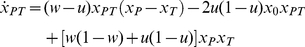
(4)where 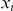 is the frequency of type 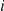, and 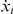 its rate of change (derivative). We now study the cultural dynamics emerging from these equations and relate it to the empirical findings considered above. We consider a generic initial condition in which all types have nonzero frequency. We note from the outset that the frequency of the preference can never increase. Indeed, writing such frequency as 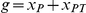 and summing equations (2) and (4), we obtain
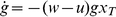
(5)which is always negative so long as 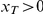 and 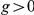. Thus this model cannot explain how a population comes to prefer a given cultural trait. We address how this can happen later in the paper. Here we study cultural dynamics assuming that the preference has reached a frequency 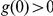 (see also Model S1).

**Figure 2:**
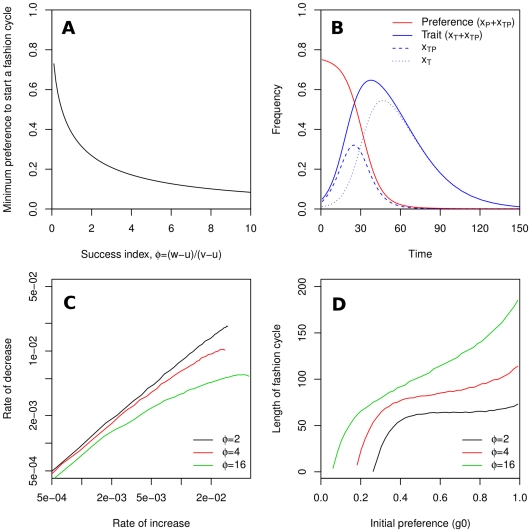
Characteristics of fashion cycles generated by Model 1 (equations 1–4). (A) Minimum initial frequency, 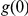 necessary to start a fashion cycle for system parameters 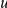, 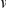, 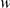 such that 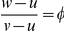. The curve has equation 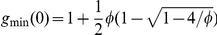 (Model S1). (B) Example of fashion cycle starting from initial preference frequency 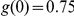 and initial trait frequency 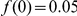, with parameters 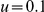, 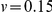, 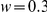. (C) Correlation between rate of increase and decrease of traits. (D) Duration of fashion cycles. The initial frequency of the preference interacts non-linearly with system parameters: only when 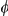 is high very long cycles can occur, given a high initial preference. Maximum trait frequency is approximately proportional to the initial preference, and does not depend strongly on 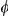 (Figure S4).

Equations (1–4) imply that the trait eventually disappears, while the preference may persist at a very low frequency (see Model S1). Fashion cycles are possible, however, because trait frequency can increase for some time before starting to drop. Let 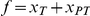 be the frequency of the trait in the population. Its dynamics, obtained summing equations (3) and (4), is
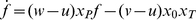
(6)Hence trait frequency increases as long as
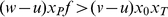
(7)This condition is easily understood noting that, on the l.h.s., 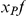 is the rate at which individuals with the preference, and lacking the trait, meet individuals with the trait, while, on the r.h.s., 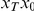 is the rate at which individuals with the trait, and lacking the preference, meet individuals without trait nor preference. In the first kind of encounters trait frequency increases with probability 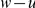, while in the second kind of encounters trait frequency decreases with probability 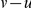. We show in Model S1 that a fashion cycle occurs provided the initial frequency 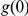 is higher than a threshold value (graphed in Figure 2A), which is a function of the combination of system parameters given by
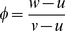
(8)If the initial preference is lower than the threshold, trait frequency steadily decreases without showing the rise-and-fall pattern characteristic of fashion cycles. The initial frequency of the trait plays no role in determining the success of the trait: even a trait that is introduced at a high frequency (e.g., through promotional sales or other marketing strategies) will disappear quickly unless it is preferred by sufficiently many individuals.

Figure 2B shows an example of the dynamics generated by this model, assuming that the frequency of the trait is initially low, 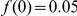, and that the preference is initially high, 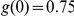. As anticipated, the preference steadily decreases, while the trait exhibits a cycle of initial diffusion and eventual abandonment. The cycle results from the superposition of two cycles, first involving the growth and decline of cultural type 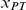 and then of type 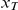. When a cycle occurs, we observe a strong correlation between the rate of increase in trait frequency and the rate of subsequent decrease (Figure 2C), mirroring empirical observations (Figure 1B).

**Figure 3:**
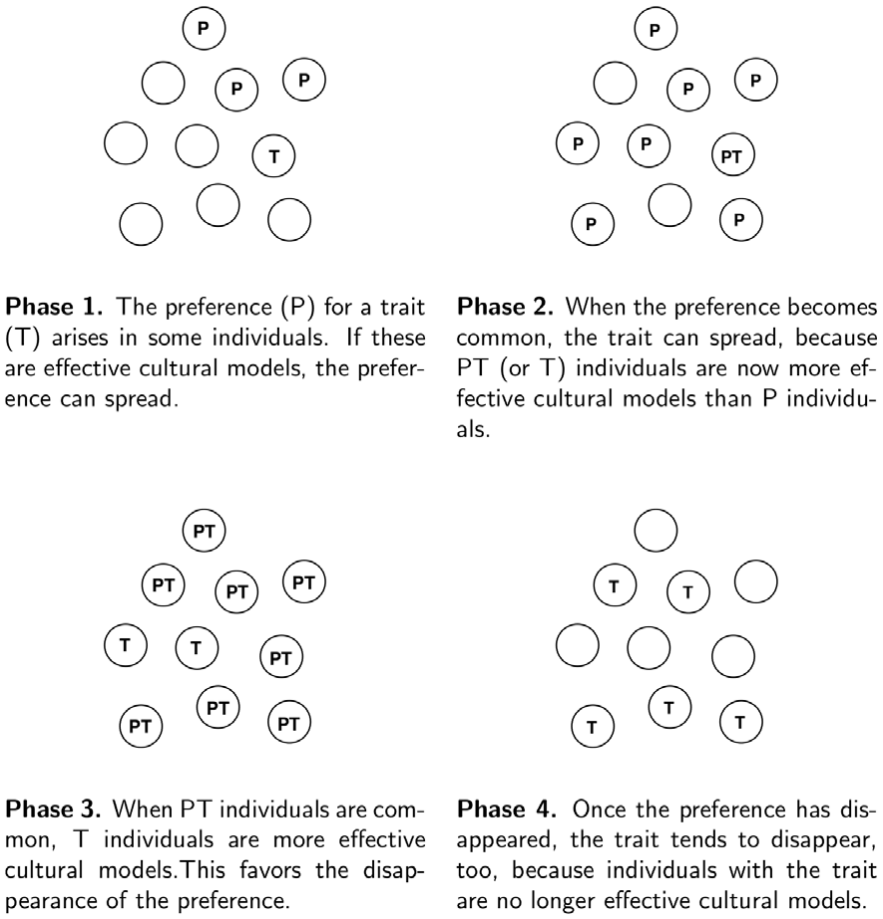
Schematic illustration of the phases of the fashion cycle of a cultural trait.

The dynamics of Model 1 (Figure 2B) suggests a theory of the fashion cycle as composed of four phases (Figure 3):

1. The preference for a trait arises in the population (Figure 3A). As already mentioned, this phase is not captured by the model above, and is studied later in the paper.
2. If the preference becomes sufficiently common, the trait itself starts to spread and 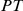 individuals, possessing both the preference and the trait, increase in frequency as the many 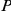 individuals meet the few 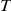 individuals (Figure 3B, and dashed line in Figure 2B). The reason is that, when the preference is common, 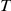 individuals are more effective cultural models than 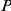 individuals, i.e., encounters between these two types result in a net flow of 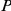 into 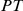. The magnitude of such flow is regulated by the difference 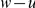 in our model.
3. When 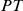 individuals become common, the situation changes as 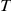 individuals gain an advantage in social interactions. Interactions between 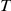 and 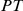, in fact, favors 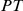 individuals becoming 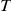 rather than vice-versa, leading to the eventual disappearance of the preference from the population (Figure 3C, and dotted line in Figure 2B).
4. Once the preference has disappeared, the trait tends to disappear, too, because individuals with the trait (both 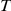 and 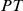) are no longer copied, and because 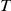 individuals eventually relinquish the trait upon meeting 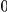 individuals (Figure 3D).

The somewhat surprising shift in the dynamics in phase 3, that explains how a fashion begins to fade, has been studied in previous work exploring what makes an individual an effective cultural model, i.e., someone who significantly influences others [22]–[24]. The basic idea is that, when a cultural trait is common, individuals with a low preference for the trait are favored as cultural models because they are less likely to copy others, and thus they display to observers a stable set of traits that can be transmitted repeatedly (see also [25], [26]). In line with this logic, 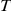 individuals increase in frequency at the expense of 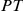 individuals, when then the latter are common, because the 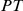 type is more likely to copy the 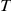 type than vice-versa.

The correlation between rates of increase and decrease of trait frequency arises because the speed at which the successive phases of the fashion cycle unfold is largely determined by the initial frequency of the preference. The key observation is that transformations between cultural types occur at rates proportional to the abundance of the cultural types themselves. Thus a large initial preference produces quickly relatively many 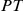 individuals, as there are many 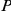 observer who can adopt the trait from 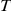 and 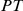 models (transition from phase 2 to phase 3 in Figure 3, or dashed blue line in Figure 2B). As 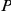 individuals turn quickly into 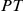, transitions from 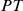 to 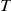 increase in frequency and quickly convert 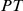 into 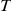 individuals (transition from phase 3 to phase 4 in Figure 3, or dotted blue line in Figure 2B), which eventually leads to the abandonment of the trait. When the initial preference is lower, all these processes occur more slowly because the level reached by the variables 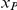, 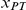, and 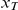 is lower.

In conclusion, this simple model shows that the cultural transmission of traits and preferences is sufficient to generate fashion cycles with intuitively appealing features, including the observed correlation between rates of increase and decrease in trait popularity. The model cannot, however, explain how preferences for traits can reach high levels, and cannot explain the observed frequency distribution of cultural traits. To address these issues, we generalize the model to include many cultural traits and preferences.

### Model 2: Many cultural traits

To describe individuals who can bear many cultural traits and preferences, we introduce the variables 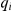 (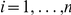) that encode whether the individual lacks (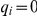) or possesses (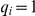) any of 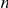 traits, and by variables 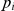 that encode the individual's preferences for the traits. Preferences range continuously from 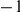 (strong dislike) to 1 (strong liking). Our core assumption is that an observer's probability to copy a cultural model is an increasing function of how much the observer prefers the model's traits. We compute the overall preference of observer 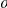 for model 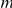 as
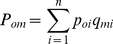
(9)and we define the probability that cultural transmission occurs from the model to the observer as
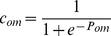
(10)This expression ranges from near 0, when the model has many traits that the observer dislikes (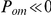), to near 1, when the model has many preferred traits (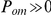). When the observer is indifferent to the model's traits (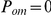), the probability of cultural transmission is 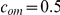. When an observer copies a model, she chooses a trait-preference randomly, then copies each member of the pair independently of the other with probability 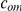. Equations (9) and (10) are similar to successful models of human decision making, such as discrete choice models in econometry [27] and neural network models of behavior [28], in which decisions arise from individuals attributing different weights (preferences) to pieces of information from their environment.

Model 2 reduces to Model 1 when there is only one cultural trait and preferences only have two values. For example, choosing −1 and 1 as possible preferences results in 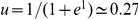 for the probability that an observer without the preference copies the model, and 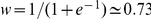 for the probability that an observer with the preference copies a model with the trait. In Model 2 we do not need to assume a special rule for interactions between observers with the trait, but lacking the preference, and models with neither trait nor preference (parameter 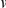 in Model 1). Rather, when many traits are present, those for which preferences are low are abandoned spontaneously as a result of competition with other traits for the opportunity of being copied.

### Neutral model and status model

We simulate a neutral model of cultural evolution that mirrors closely existing ones [4], but allows individuals to interact many times during their life [25] and to accumulate cultural traits [29], [30]. In this model there are no preferences for cultural traits, hence we fix the probability of cultural transmission at 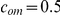. When an observer copies a model, she copies a randomly chosen trait (the observer can also abandon a trait, if the trait randomly chosen is possessed by the observer but not by the model).

We also simulate a model of cultural transmission based on status. Individual are endowed with an additional variable that describes their social status (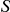). Cultural transmission occurs with a constant probability 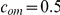 as in the neutral model, but the outcome depends on the relative status of observer and model. If 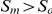, the observer copies from the model a randomly chosen trait. If 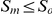, the observer abandons a randomly chosen trait possessed by both observer and model. The status of newborn individuals is assigned randomly. We also simulated alternative status models, either with binary status (95% of low-status individuals and 5% “high-status”) or varying the threshold for abandoning or copying traits, with qualitatively similar results not reported here.

## Results

**Figure 4:**
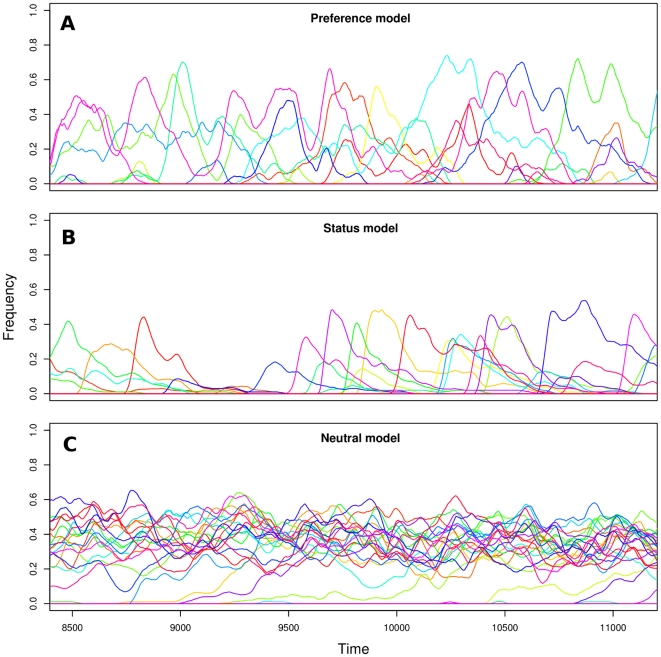
Changes of trait frequency over time in the multi-trait models of fashion. We simulate 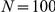 individuals interacting randomly in discrete time steps. Cultural traits are continuously introduced with a probability 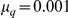 per individual per time step (a new trait is introduced, on average, every 10 time steps). Individuals may spontaneously change their preferences for existing traits, resetting them to random values between −1 and 1 at a rate of 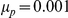 per individual per time step. Each individual has a probability of dying of 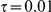 per time step (average lifetime is 100 time steps). A dying individual is replaced by a naive individual who possesses no cultural traits (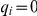, 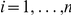) and is maximally open to learning for others (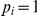, 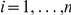). Simulations last 20,000 time steps (only 3,000 are shown in the figure, and a maximum of 40 traits is shown to maintain legibility).

**Figure 5:**
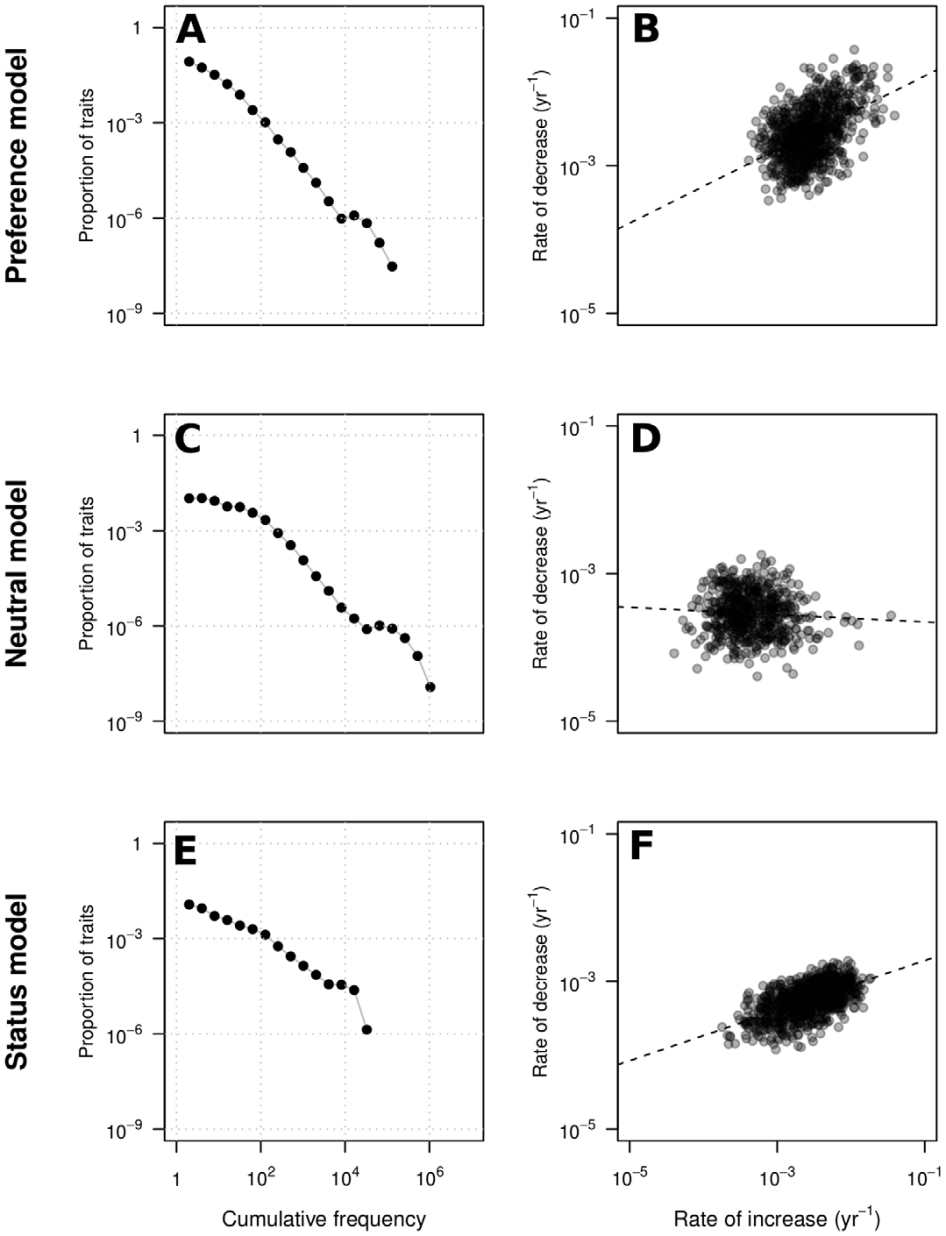
Characteristics of cultural dynamics in the multi-trait models of fashion. (A,C,E) Cumulative frequencies of traits (cf. Figure 1A for empirical data). (B,D,F) Correlations between rates of increase and decrease of traits (cf. Figure 1B for empirical data): preference model (Pearson's 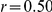, 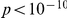, 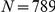) neutral model (Pearson's 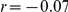, 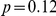, 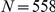), and status model (Pearson's 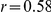, 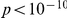, 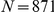). Data from 5 simulations with the same parameters as in Figure 4. Simulated time steps have been converted to years assuming an average lifetime of 70 years.

The preference, neutral and status models all exhibit dynamics in which trait frequencies change with time, and even traits that reach high frequencies eventually disappear (Figure 4). The preference and neutral models generate realistic frequency distributions, while the status model appears incapable of generating traits that persist at high frequency for a long time (Figure 1A and Figure 5A,C,E, and Figure S5 for trait lifespan distributions). The reason is that as soon as a trait becomes common, high-status individuals abandon it, which in turns triggers abandonment from low-status individuals. Thus the status model may account for brief fads, but not for “classic” styles such as the four-in-hand tie knot popular since the early 20^th^ century [31], or English names such as Mary and John [32].

The preference and status models exhibit correlations between the rate of increase and decrease of traits similar to those observed in empirical data (Figure 1B and Figure 5B,D,F). Such correlations do not exist in the neutral model, because the frequency of a trait at time 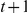 depends solely on the frequency at time 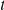 and not on the history of the trait. In the preference model, on the other hand, the frequency of a trait at time 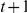 depends on the frequency of the trait at time 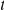 *and* on the preference value at time 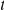 (cf. Figure 2B).

**Figure 6:**
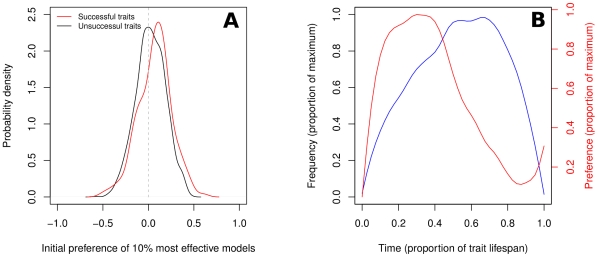
Dynamics of preference-trait coevolution in Model 2. (A) Initial preferences of the 10% most effective cultural models at the start of a trait's lifetime, for traits that will be successful (red, mean = 0.08) or unsuccessful (black, mean = 0.02, 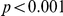, two-tailed Wilcoxon rank-sum test, the graph shows estimated probability densities). Traits are considered successful if they reach a frequency of at least 0.1. For successful traits, initial preference is calculated as the average preference from the time a trait is introduced until the trait reaches 0.1 frequency. For unsuccessful traits, initial preference is calculated as the average over the mean time required for successful traits to reach 0.1 frequency. (B) Black line: Trait frequency as fraction of the maximum frequency. Red line: Average preference as fraction of the maximum preference. The average is computed over all traits reaching at least a frequency of 0.1, and lasting in the population for at least 50 time steps. Time is measured as proportion of the trait's lifetime. Simulation parameters as in Figure 4.

The many-trait preference model (Model 2) addresses the main shortcoming of the model with one trait (Model 1), showing that the preference for a trait can spread if it happens to be associated with effective cultural models (Figure 6A). As these individuals are copied more often than others, the preference spreads in the population. After a preference has been established, fashion cycles in Model 2 follow the same logic as in Model 1. Figure 6B shows that a trait increases in frequency when the associated preference has increased, and that the preference starts falling as the trait becomes common (cf. Figure 2B; see Figure S6 for examples of the preference-frequency dynamics of individual traits). Cross-correlation analysis of trait and preference dynamics shows that preferences anticipate frequencies by, on average, 20% of a trait's lifetime, with an average cross-correlation of 0.60 (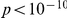, 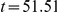, 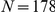, two-tailed one-sample 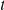 test).

## Discussion

We have shown that the social transmission of preferences for cultural traits is sufficient to generate realistic fashion cycles, resulting in a better fit with empirical data than the neutral and status models of cultural change (cf. Figures 1 and 5).

Our model also overcomes theoretical shortcomings of previous models. In respect to the neutral model, it recognizes that people have preferences for cultural traits and individuals and that those preferences influence the copying process [19]–[21]. In respect to the status model, it suggests how social status can emerge from cultural evolution itself. In our model, influential individuals are those who possess many traits that others prefer and, at the same time, have low preferences for widespread traits. This echoes the concept of “high-status” in status models (high-status individuals are copied by others, but do not themselves copy others), without assuming either a pre-existent social stratification or anti-conformist behavior in high-status individuals. In the preference model, moreover, an individual's status can change if traits or preferences in the population change.

Alternative, more elaborated, versions of the status model could produce results different from the ones shown here, and possibly a better fit of the empirical data. For example, it has been suggested that individuals adopt cultural traits to signal their identity and differentiate themselves from individuals of other social groups, without necessarily implying a “vertical” (high/low) status hierarchy [33], [34]. Our goal here is certainly not to deny the importance of status or identity signaling, but to show, as explained above, that at least certain features of status-driven cultural dynamics can be reproduced within a more parsimonious set-up.

Finally, it is worth to note that the quantity 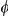 in equation (8) can be considered a “success index” akin to genetic fitness, in that it allows to predict whether a trait will spread. While it is impossible to define a generally valid success index for cultural evolution [25], ratios similar to 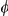 may predict the success of cultural traits when individuals have many opportunities for social learning [22], [23], [25]. Numerators in such ratios measure the ease with which a trait is transmitted. Here, the difference 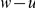 measures the advantage, as cultural models, of individuals with the trait. More broadly, transmissibility may relate to specific characteristics of traits that make them easy to learn or otherwise acquire (e.g., affordable or widely marketed clothing items), or to their psychological appeal (resulting in what is often referred in cultural evolution literature as content-biased transmission, see [35]), and so on. For example, it has been shown that traits that elicit emotional reactions in general [36], or particular emotion like disgust [37] tend to be transmitted more than traits that do not.

On the other hand, since individuals have many occasions to learn new traits and replace the existing ones, denominators in indices of cultural success measure the resistance of traits in being relinquished. Here, the difference 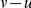 measures how easily individuals lacking the preference abandon a trait. This suggests that traits that are particularly memorable, useful, or otherwise durable (e.g., tattoos) should enjoy an advantage in cultural evolution. Here we have shown that such ratios are not only theoretically important, but yield insight into actual cultural processes.

## Supporting Information

Model S1 **Derivation and analysis of Model 1.**
(PDF)

Figure S1
**Goodness of simplified model.** Absolute difference between trait frequency (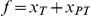) according to the full Model 1 (equations 1–4 in the main text) and the simplified model in equations (6,7,9) as a function of initial preference, 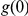, and for different combinations of 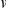 and 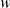 parameters (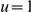). Trait frequency in the simplified model lies within 2% of the frequency given by the full model.(TIF)

Figure S2
**Sample model trajectories.** Sample model trajectories in the 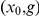 plane of the simplified system in equations 6–7 (Model S1), for different initial frequencies of the preference, 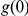, and initial frequency of the trait 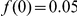. Trajectories start at the closed circle and end at the open diamond. The dashed line is the line 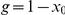, which delimits the state space together with the lines 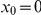 and 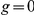. The red line is the locus of all starting conditions with 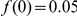 (assuming the trait and the preference are initially distributed independently). The dotted lines are trajectories of the full system, equations 1–4 in the main text, showing the quality of our approximation.(TIF)

Figure S3
**Equilibrium values of preferences.** Frequency of the preference for a trait at the end of a fashion cycle (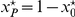 in equation 15 (Model S1) as a function of initial frequency of the preference, 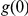. All parameters as in Figure S2.(TIF)

Figure S4
**Maximum frequency attained during a trait's fashion cycle.** Maximum frequency attained during a trait's fashion cycle, as a function of initial preference, 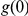, and system parameters, 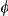. Initial frequency is 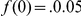.(TIF)

Figure S5 **Distribution of trait lifespans in the multi-trait models of fashion.** Distribution of trait lifespans for the preference, status and neutral models for the simulations described in the main text.
(TIF)

Figure S6 **Examples of frequency-preference dynamics in simulations of the multi-trait preference model.** (A) The preference (red) for a trait rises in the population, which causes a rise in frequency (blue). As the trait becomes common, the preference falls and eventually the trait declines in frequency. See main text for discussion. (B) Another example of the same dynamics, showing that the latency between rise in preference and rise in frequency may be long. (C) A trait undergoing multiple fashion cycles. Trait revival is possible by either chance fluctuation or because effective models adopt the trait again. (D) A trait that remains popular for some time, despite not being preferred. This may happen because a common trait is likely to be possessed by successful cultural models, hence it can be copied even if it does not contribute to the model's success. A real-life example may be common names such as George, who may not be perceived as particularly catchy but is nevertheless associated with successful individuals such as George Washington, George Harrison, or George Clooney.
(TIF)

Table S1 Model 1 transitions.
(PDF)
