# Supplementary material for: The Logic of Fashion Cycles
Source: PLoS One. 2012 Mar 7;7(3):e32541. doi: 10.1371/journal.pone.0032541 (PMC3296716; doi:10.1371/journal.pone.0032541)
Supplement: Model S1 — Derivation and analysis of Model 1. (PDF) [file pone.0032541.s001.pdf]

Supporting information for "The logic of fashion cycles," A. Acerbi, S. Ghirlanda, and M. Enquist.

Model S1

Derivation

Equations (1–4) in the main text are derived considering the possible outcomes of interactions of observers of type i with models of type j, with $i, j \in \{0, P, T, PT\}$. According to the rules given in the main text, the probability that an observer of type i copies a model of type j is given by the entries in the following array:

	Model:			
Observer:	0	P	T	PT
0		u	u	u
P	u		$\boldsymbol{w}$	$\boldsymbol{w}$
T	$\boldsymbol{v}$	u		u
PT	u	u	$\boldsymbol{w}$	

where the entries in boldface reflect our assumptions on the effect of preference and lack of preference on cultural transmission. Interactions of models and observers of the same type are not considered as they do change the frequency of types.

Transition rates between types are constructed as follows. Suppose, for instance, that an observer of type PT meets a model of type T. Trait and preference are copied independently with probability w. Thus with probability $w(1-w)$ the observer copies the model's preference value but not its trait value, resulting in the observer changing from PT to T. With probability $w(1-w)$ the observer copies the model's trait value but not its preference value, which results in no change in the observer's type. With probability w^2 the observer copies both the trait and preference values, resulting in a change from type PT to T. Hence in interactions between PT observers and T models there is overall a probability $w(1-w) + w^2 = w$ that the observer changes from PT to T, and a probability $1 - w$ that the observer does not change. Since encounters between PT and T occur at a rate of $x_{PT}x_T$, the overall rate at which such transitions occur is $w\, x_{PT}x_T$. Table S1 shows the rates of all possible transitions, calculated in this same way (the one just calculated is entry 20). Equations (1–4) in the main text

follow from calculating the net effect of these transitions as follows:

$$\begin{aligned} \dot{x}_0 &= -(1:5)+6+7+11+12+16 \quad (1)\\ \dot{x}_P &= -(6:10)+1+3+14+18+19 \quad (2)\\ \dot{x}_T &= -(11:15)+2+4+8+17+20 \quad (3)\\ \dot{x}_{PT} &= -(16:20)+5+9+10+13+15 \quad (4) \end{aligned}$$

where numbers refer to lines in Table S1 and $a:b$ indicates the range from a to b inclusive.

Analysis

We study the model using a mix of analytical and numerical methods as follows. We noticed numerically that the equation for $\dot{x_0}$ (equation 1 in the main text) can be simplified as

$$\dot{x}_0 = (v-u)\,x_0 x_T \quad (5)$$

as the sum of the two other terms is between 10 and 100 times smaller than $(v-u)\,x_0 x_T$ over a range of initial conditions and parameter values (Figure S1). This simplification allows us to write a closed system for x_0 and $g = x_P + x_{PT}$:

$$\dot{x}_0 = (v-u)\,x_0\,(1-g-x_0) \quad (6)$$

$$\dot{g} = -(w-u)\,g\,(1-g-x_0) \quad (7)$$

where we have eliminated x_T through the identity

$$x_T + x_0 + g = 1 \quad (8)$$

The description of the system is completed by the equation for the trait frequency $f = x_T + x_{PT}$ (equation 6 in the main text), which in terms of the variables x_0, g, and f is rewritten as:

$$\dot{f} = -(v-u)\,x_0(1-g-x_0) - (w-u)\,f(1-f-x_0) \quad (9)$$

Equilibria

We denote equilibrium values by a superscript *. Equilibria of equations (6–7) are of the form

$$x_0^\star + g^\star = x_0^\star + x_P^\star + x_{PT}^\star = 1 \quad (10)$$

implying

$$x_T^\star = 0 \tag{11}$$

Given equation (10) and the equilibrium condition $\dot{f} = 0$, equation (9 implies

$$f^\star(1 - f^\star - x_0^\star) = 0 \tag{12}$$

which, using equation (11) and expanding $f^\star = x_T^\star + x_{PT}^\star$ yields

$$x_{PT}^\star x_P^\star = 0 \tag{13}$$

Implying that either $x_P^\star = 0$ or $x_{PT}^\star = 0$, or both. Equation (4) in the main text, however, implies $x_{PT}^\star = 0$ for all $x_0^\star > 0$. In conclusion, equilibria of the model are of the form:

$$x_0^\star + x_P^\star = 1 \qquad x_{PT}^\star = x_T^\star = 0 \tag{14}$$

hence the trait cannot persist in the population, but the preference can. The following analysis characterizes system trajectories and shows that $x_P^\star$ is generally small.

System trajectories

Because x_0 and g are frequencies, they must be ≥ 0 and must sum to a number less than 1. Thus equations (6) and (7) hold in the triangle defined by $x_0 \geq 0$, $g \geq 0$, $1 - x_0 - g \geq 0$. In its interior, $\dot{g} < 0$ and $\dot{x}_0 > 0$ always. Taking the ratio of equations 6 and 7 we obtain a differential equation for the shape of the system trajectory as a curve $g = g(x_0)$:

$$\frac{dg(x_0)}{dx_0} = \frac{dg}{dt}\frac{dt}{dx_0} = -\frac{w-u}{v-u}\frac{g}{x_0} \tag{15}$$

The combination of parameters $(w-u)/(v-u)$ recurs often, hence we define

$$\phi = \frac{w-u}{v-u} \tag{16}$$

The solution of (15) is thus

$$g(x_0) = C\, x_0^{-\phi} \tag{17}$$

where the constant C is set from initial conditions as

$$C = g(0)\,(x_0(0))^\phi \tag{18}$$

Examples of these trajectories are in Figure S2 given the initial condition $f(0) = 0.05$ and varying $g(0)$ as indicated. All trajectories end on the line $g = 1 - x_0$, meaning that $x_T^\star = 0$; as showed in the previous section, $x_{PT}^\star = 0$ as well. The value of $x_0^\star$, and thus of $x_P^\star = 1 - x_0^\star$ is the solution of

$$C(x_0^\star)^{-\phi} + x_0^\star - 1 = 0 \tag{19}$$

with the highest value (there are two solutions, as it is apparent from Figure S2). This equation usually yields very low values of x_P (Figure S3).

Existence of fashion cycles

We have established in the previous sections that all fashions eventually die in this model. A fashion cycle occurs when, before disappearing, a trait initially increases in frequency. We determine here, for given parameters u, v, and w the minimum initial frequency of the preference, $g_{\min}(0)$, for a cycle to occur.

Given initial conditions $f(0)$ and $g(0)$ the condition for f to initially increase is, from equation (9):

$$-(v-u)\,x_0(0)(1-g(0)-x_0(0)) + (w-u)\,f(0)(1-f(0)-x_0(0)) > 0 \tag{20}$$

Assuming that trait and preference are initially distributed independently, implying the initial conditions $x_{PT}(0) = f(0)g(0)$, $x_T(0) = f(0)(1-g(0))$, and $x_P(0) = g(0)(1-f(0))$, we have

$$x_0(0) = 1 - f(0) - g(0) + f(0)g(0) \tag{21}$$

Substituting this expression in equation (20) we get the condition

$$g_{\min}^2(0) - (2+\phi)g_{\min}(0) + 1 < 0 \tag{22}$$

where $\phi = \frac{w-u}{v-u}$ as defined in equation (16). The solution is

$$g_{\min}(0) > 1 + \frac{\phi}{2}\left(1 - \sqrt{1 + \frac{4}{\phi}}\right) \tag{23}$$

(The second solution of the quadratic equation (22) is of no interest as it is always > 1). Note that this condition does not rely on the simplified dynamics of x_0 in (6) and, moreover, it is independent of $f(0)$. Thus, for a given value of ϕ, the initial preference determines whether a fashion cycle occurs irrespective of the initial frequency of the trait.

Maximum frequency

A differential equation for trait frequency as a function of x_0 is obtained proceeding as in equation (15):

$$\frac{df(x_0)}{dx_0} = -1 + \phi \frac{f(1 - f - x_0)}{x_0(1 - g(x_0) - x_0)} \tag{24}$$

This equation, however, does not appear to have a closed form solution as equation (15), hence it is not possible to determine analytically the maximum frequency attained. It is possible to obtain lower and upper bounds through the identity $x_T \leq f \leq x_T + g$, accurate to about 10% for small $g(0)$ to near-perfect for large $g(0)$, but the formulae are not telling and we prefer to present numerical results. Figure S5 shows that the maximum frequency grows approximately linearly with $g(0)$, and is not greatly influenced by ϕ.
