# Supplementary figures and images for: The Logic of Fashion Cycles
Source: PLoS One. 2012 Mar 7;7(3):e32541. doi: 10.1371/journal.pone.0032541 (PMC3296716; doi:10.1371/journal.pone.0032541)

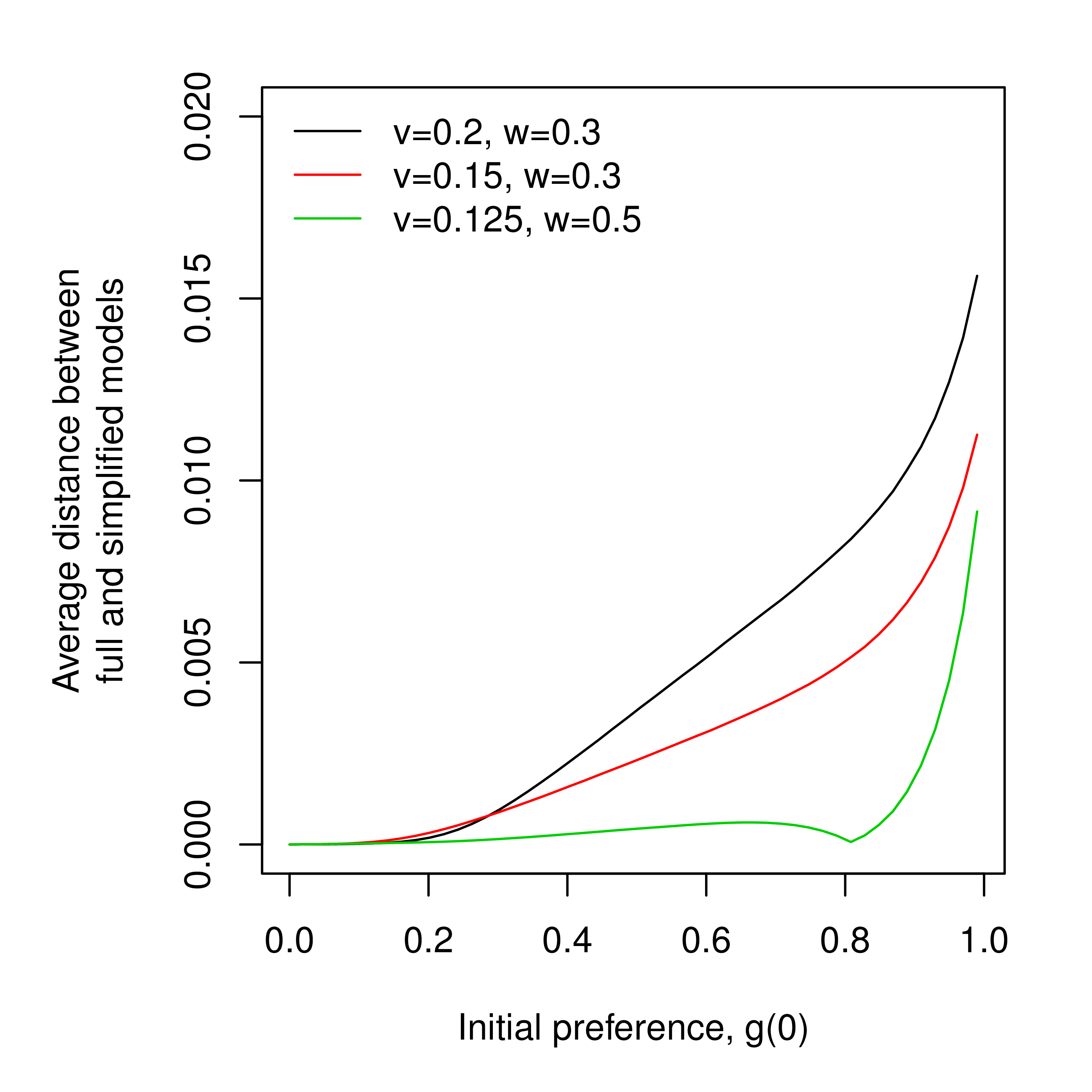

Supplement: Figure S1 — Goodness of simplified model. Absolute difference between trait frequency () according to the full Model 1 (equations 1–4 in the main text) and the simplified model in equations (6,7,9) as a function of initial preference, , and for different combinations of and parameters (). Trait frequency in the simplified model lies within 2% of the frequency given by the full model. (TIF) [file pone.0032541.s002.tif]

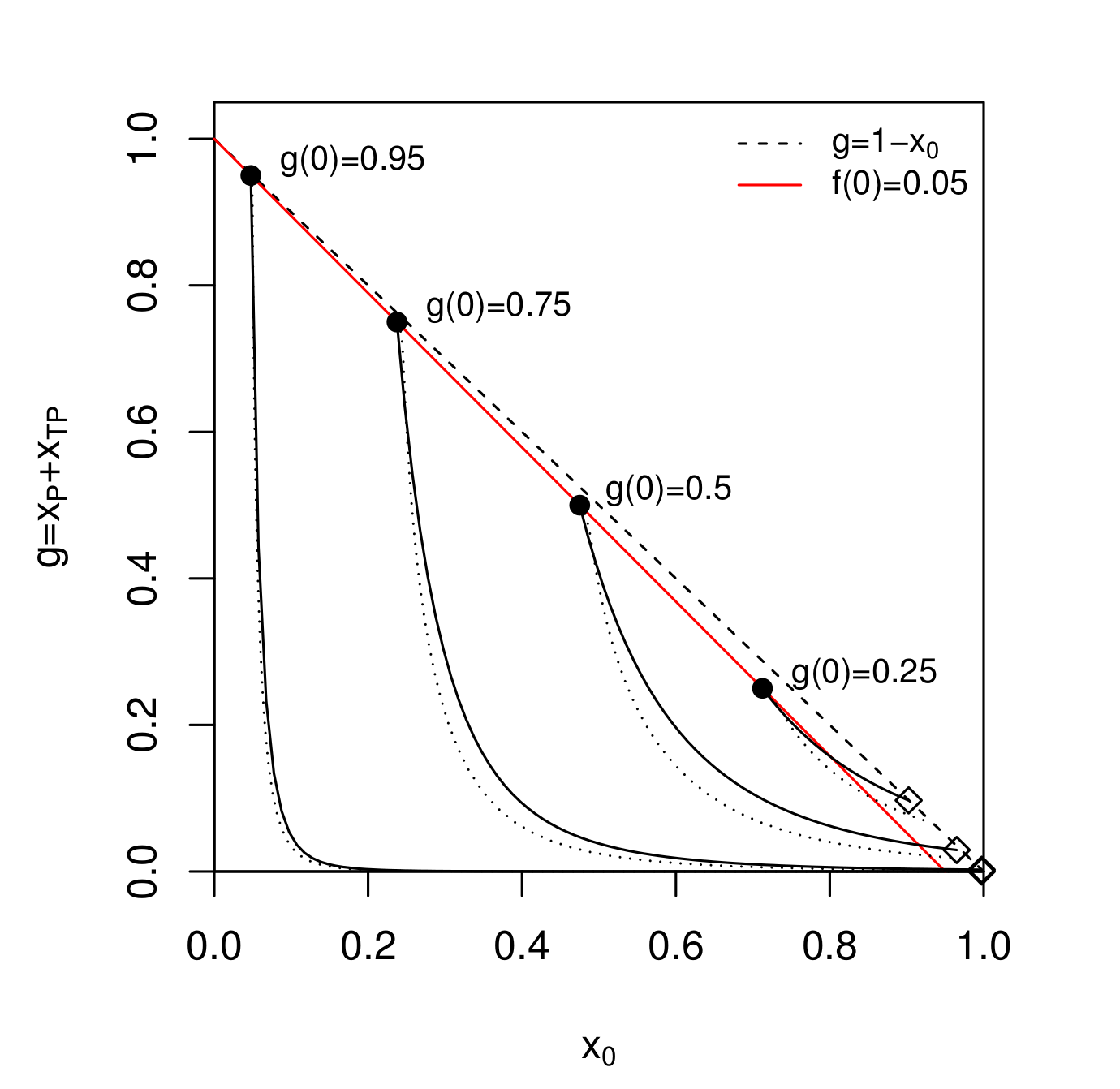

Supplement: Figure S2 — Sample model trajectories. Sample model trajectories in the plane of the simplified system in equations 6–7 (Model S1), for different initial frequencies of the preference, , and initial frequency of the trait . Trajectories start at the closed circle and end at the open diamond. The dashed line is the line , which delimits the state space together with the lines and . The red line is the locus of all starting conditions with (assuming the trait and the preference are initially distributed independently). The dotted lines are trajectories of the full system, equations 1–4 in the main text, showing the quality of our approximation. (TIF) [file pone.0032541.s003.tif]

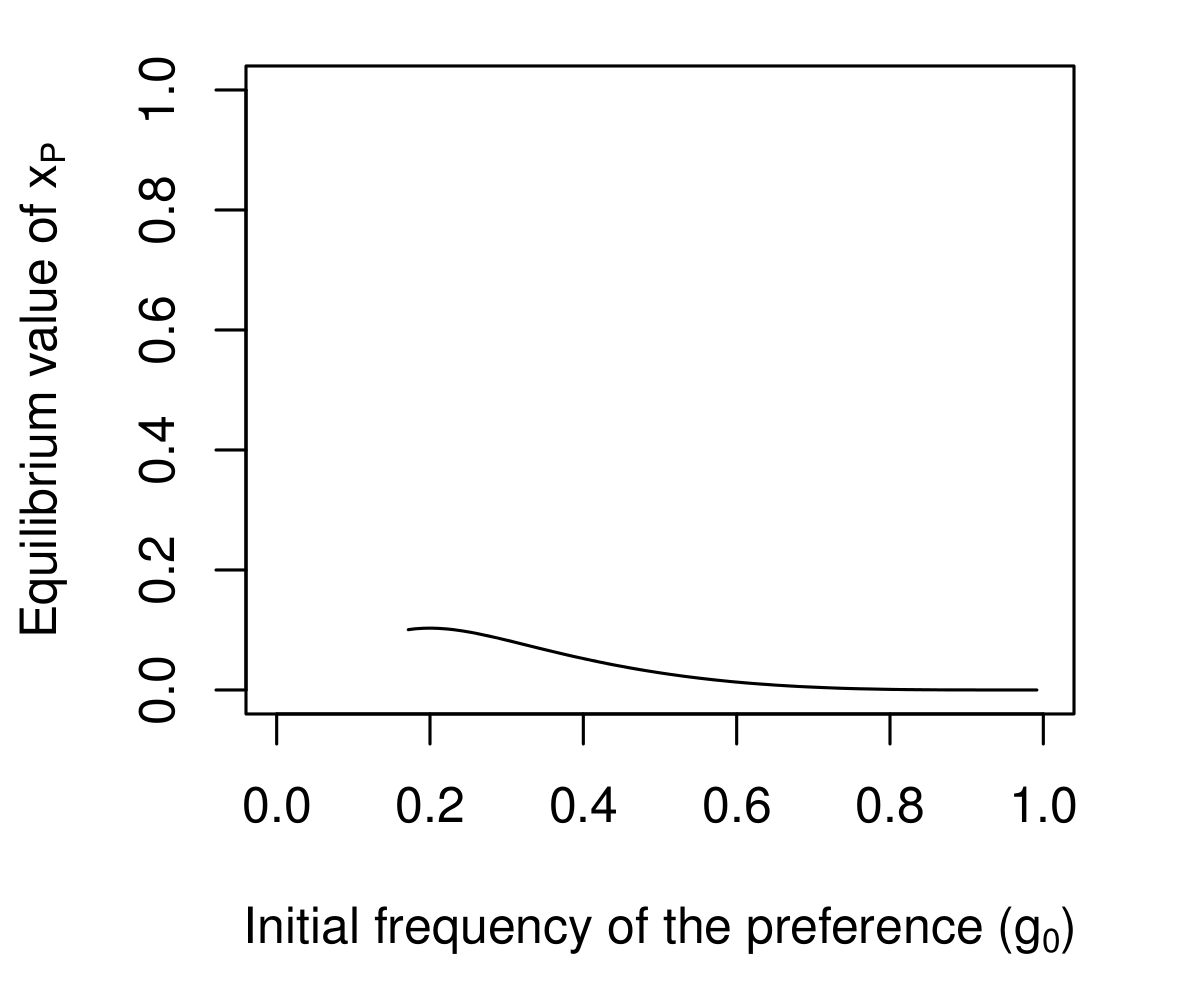

Supplement: Figure S3 — Equilibrium values of preferences. Frequency of the preference for a trait at the end of a fashion cycle ( in equation 15 (Model S1) as a function of initial frequency of the preference, . All parameters as in Figure S2. (TIF) [file pone.0032541.s004.tif]

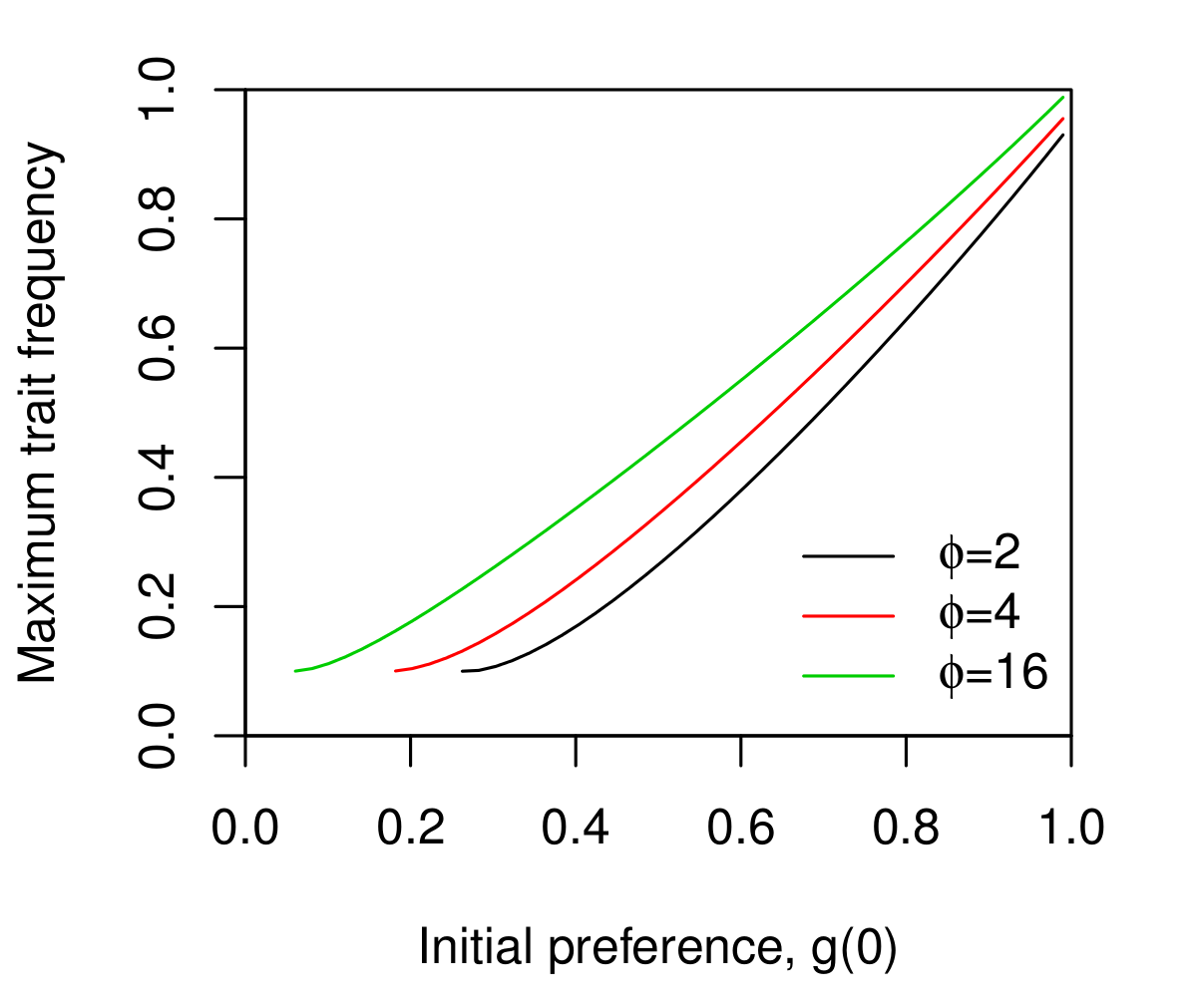

Supplement: Figure S4 — Maximum frequency attained during a trait's fashion cycle. Maximum frequency attained during a trait's fashion cycle, as a function of initial preference, , and system parameters, . Initial frequency is . (TIF) [file pone.0032541.s005.tif]

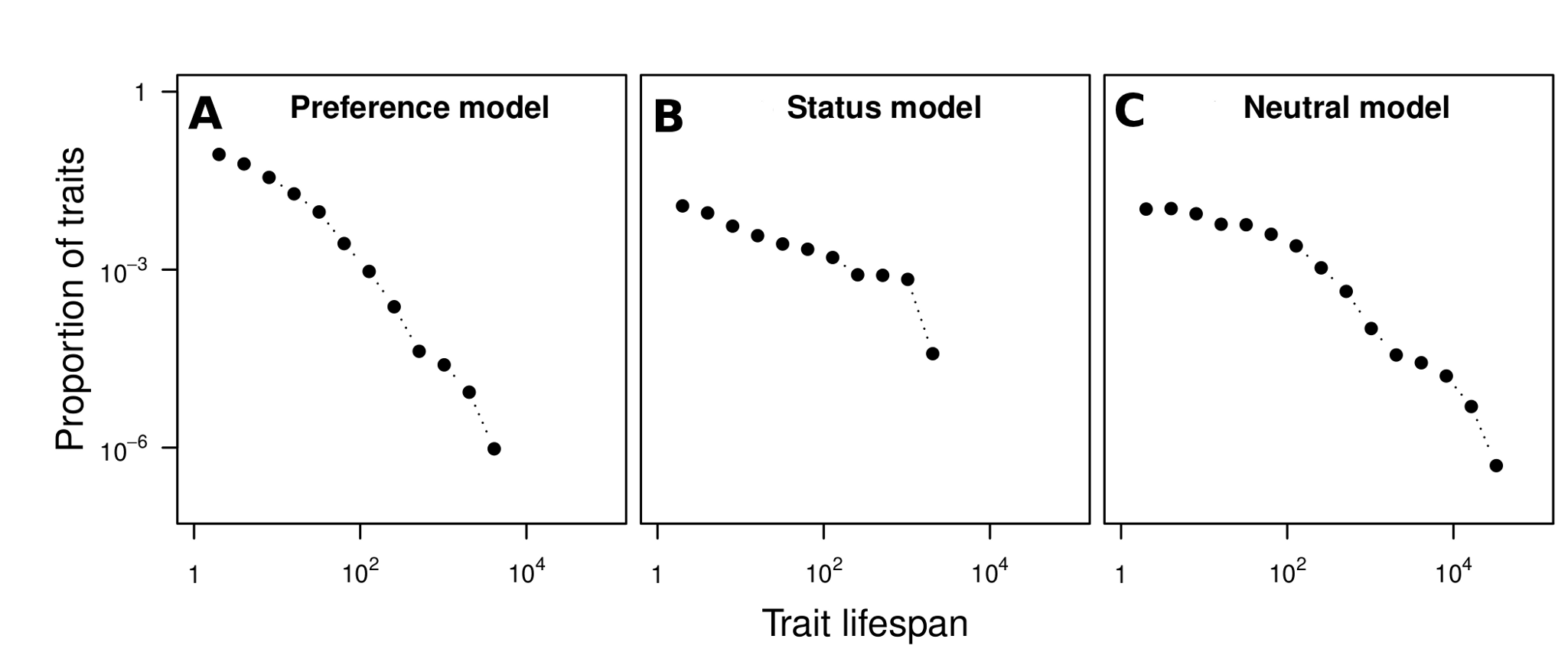

Supplement: Figure S5 — Distribution of trait lifespans in the multi-trait models of fashion. Distribution of trait lifespans for the preference, status and neutral models for the simulations described in the main text. (TIF) [file pone.0032541.s006.tif]

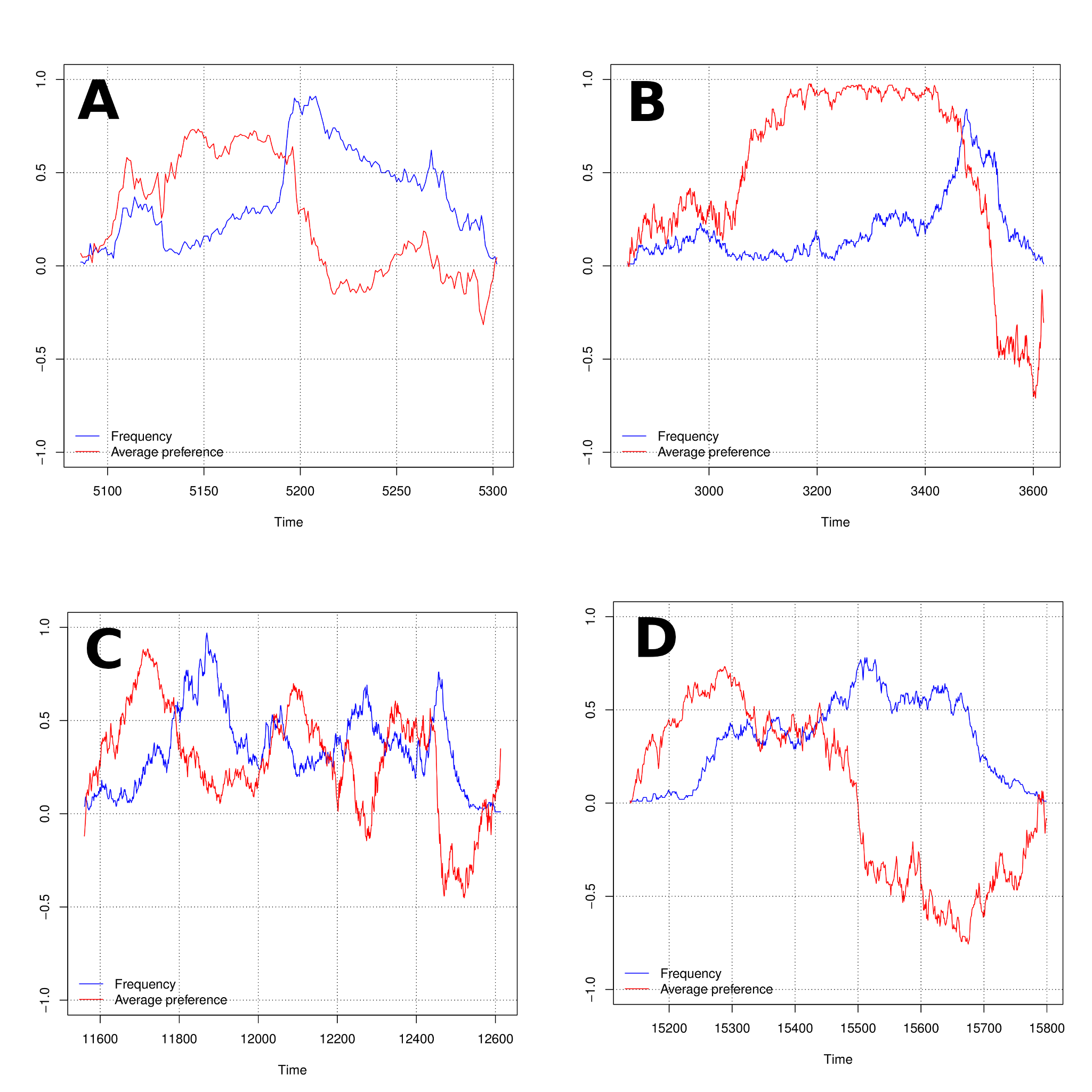

Supplement: Figure S6 — Examples of frequency-preference dynamics in simulations of the multi-trait preference model. (A) The preference (red) for a trait rises in the population, which causes a rise in frequency (blue). As the trait becomes common, the preference falls and eventually the trait declines in frequency. See main text for discussion. (B) Another example of the same dynamics, showing that the latency between rise in preference and rise in frequency may be long. (C) A trait undergoing multiple fashion cycles. Trait revival is possible by either chance fluctuation or because effective models adopt the trait again. (D) A trait that remains popular for some time, despite not being preferred. This may happen because a common trait is likely to be possessed by successful cultural models, hence it can be copied even if it does not contribute to the model's success. A real-life example may be common names such as George, who may not be perceived as particularly catchy but is nevertheless associated with successful individuals such as George Washington, George Harrison, or George Clooney. (TIF) [file pone.0032541.s007.tif]
