# Supplementary material for: The Logic of Fashion Cycles
Source: PLoS One. 2012 Mar 7;7(3):e32541. doi: 10.1371/journal.pone.0032541 (PMC3296716; doi:10.1371/journal.pone.0032541)
Supplement: Table S1 — Model 1 transitions. (PDF) [file pone.0032541.s008.pdf]

	Observer	Model	Outcome	Rate
1.	0	P	P	$u\, x_0 x_P$
2.	0	T	T	$u\, x_0 x_T$
3.	0	PT	P	$u(1-u)\, x_0 x_{PT}$
4.	0	PT	T	$u(1-u)\, x_0 x_{PT}$
5.	0	PT	PT	$u^2\, x_0 x_{PT}$
6.	P	0	0	$u\, x_P x_0$
7.	P	T	0	$w(1-w)\, x_P x_T$
8.	P	T	T	$w^2\, x_P x_T$
9.	P	T	PT	$w(1-w)\, x_P x_T$
10.	P	PT	PT	$w\, x_P x_{PT}$
11.	T	0	0	$v\, x_0 x_T$
12.	T	P	0	$u(1-u)\, x_P x_T$
13.	T	P	PT	$u(1-u)\, x P x_T$
14.	T	P	P	$u^2\, x_T x_P$
15.	T	PT	PT	$u\, x_T x_{PT}$
16.	PT	0	0	$u^2\, x_0 x_{PT}$
17.	PT	0	T	$u(1-u)\, x_0 x_{PT}$
18.	PT	0	P	$u(1-u)\, x_0 x_{PT}$
19.	PT	P	P	$u\, x_P x_{PT}$
20.	PT	T	T	$w\, x_T x_{PT}$
